# Identification of a 4-Deoxy-l-erythro-5-hexoseulose Uronic Acid Reductase, FlRed, in an Alginolytic Bacterium *Flavobacterium* sp. Strain UMI-01

**DOI:** 10.3390/md13010493

**Published:** 2015-01-16

**Authors:** Akira Inoue, Ryuji Nishiyama, Shogo Mochizuki, Takao Ojima

**Affiliations:** Laboratory of Marine Biotechnology and Microbiology, Faculty of Fisheries Sciences, Hokkaido University, Hakodate, Hokkaido 041-8611, Japan; E-Mails: inouea21@fish.hokudai.ac.jp (A.I.); nsym2480@ec.hokudai.ac.jp (R.N.); mochizuki1227@ec.fish.hokudai.ac.jp (S.M.)

**Keywords:** alginate metabolism, *Flavobacterium*, alginolytic gene, DEH reductase, SDR, NADH

## Abstract

In alginate-assimilating bacteria, alginate is depolymerized to unsaturated monosaccharide by the actions of endolytic and exolytic alginate lyases (EC 4.2.2.3 and EC 4.2.2.11). The monosaccharide is non-enzymatically converted to 4-deoxy-l-erythro-5-hexoseulose uronic acid (DEH), then reduced to 2-keto-3-deoxy-d-gluconate (KDG) by a specific reductase, and metabolized through the Entner–Doudoroff pathway. Recently, the NADPH-dependent reductase A1-R that belongs to short-chain dehydrogenases/reductases (SDR) superfamily was identified as the DEH-reductase in *Sphingomonas* sp. A1. We have subsequently noticed that an SDR-like enzyme gene, *flred*, occurred in the genome of an alginolytic bacterium *Flavobacterium* sp. strain UMI-01. In the present study, we report on the deduced amino-acid sequence of *flred* and DEH-reducing activity of recombinant FlRed. The deduced amino-acid sequence of *flred* comprised 254 residues and showed 34% amino-acid identities to that of A1-R from *Sphingomonas* sp. A1 and 80%–88% to those of SDR-like enzymes from several alginolytic bacteria. Common sequence motifs of SDR-superfamily enzymes, e.g., the catalytic tetrad Asn-Lys-Tyr-Ser and the cofactor-binding sequence Thr-Gly-x-x-x-Gly-x-Gly in Rossmann fold, were completely conserved in FlRed. On the other hand, an Arg residue that determined the NADPH-specificity of *Sphingomonas* A1-R was replaced by Glu in FlRed. Thus, we investigated cofactor-preference of FlRed using a recombinant enzyme. As a result, the recombinant FlRed (recFlRed) was found to show high specificity to NADH. recFlRed exhibited practically no activity toward variety of aldehyde, ketone, keto ester, keto acid and aldose substrates except for DEH. On the basis of these results, we conclude that FlRed is the NADH-dependent DEH-specific SDR of *Flavobacterium* sp. strain UMI-01.

## 1. Introduction

Alginate is a viscous and gel-forming polysaccharide comprising β-d-mannuronic acid (M) and α-l-guluronic acid (G), which form poly (M), poly (G) and random (MG) blocks in the alginate polymer [1,2,3,4]. This polysaccharide is produced by brown seaweeds and certain bacteria; the seaweeds’ alginate has been widely used for food and pharmaceutical materials such as viscosifier, stabilizer, impression material, gelling agent, *etc.* [2,4]. Partially depolymerized alginate has also been recognized as a functional material since it exhibits various biological activities, e.g., promotion of root growth in higher plants [5,6,7], acceleration of growth rate of *Bifidobacterium* sp. [8], promotion of penicillin production in *Penicillium chrysogenum* [9], stimulation of proliferation of endothelial cells [10], and lowering blood pressure in human [11,12]. Further, an end product of alginate lyases (EC 4.2.2.3, EC 4.2.2.11), *i.e.*, 4-deoxy-l-erythro-5-hexoseulose uronic acid (DEH), was recently used as a carbon source for ethanol fermentation with genetically modified microorganisms [13,14,15]. This implies that alginate may become available as a biomass for bioethanol production along with molasses, starch and cellulosic biomasses. Indeed, cultivation of brown seaweeds does not need vast land, water and fertilizer, and uses of seaweeds for ethanol fermentation will not cause serious food-fuel conflicts, which have been emerged in the uses of edible carbohydrates for the bioethanol production. To realize the production of bioethanol from alginate, efficient fermentation systems for this polysaccharide should be established through extensive understanding of its metabolic pathway.

Metabolic process of alginate in alginate-assimilating bacteria is generally explained as follows: (1) Alginate is first depolymerized by the action of endolytic and exolytic alginate lyases to unsaturated monosaccharide (unsaturated mono-uronide); (2) The monosaccharide is non-enzymatically converted to an open-chain form, DEH; (3) DEH is then reduced to 2-keto-3-deoxy-d-gluconate (KDG) by certain reductase(s) and metabolized to glyceraldehyde-3-phosphate and pyruvate via the Entner–Doudoroff pathway [16,17]. These metabolites can be converted to ethanol if pyruvate decarboxylase, alcohol dehydrogenase and reducing power (NAD(P)H) are sufficiently present in cytosol. In this context, genetic engineering for the construction of alginate-fermenting bacteria has been performed by supplementing and/or strengthening the enzymes that closely relate to alginate-metabolism and ethanol-fermentation [13,14,15]. Among the alginate-metabolic enzymes, DEH reductase appears to be a crucially important enzyme since it converts alginate-derived DEH to KDG that is metabolized via the Entner–Doudoroff pathway. Recently, the DEH-specific reductase A1-R was isolated from an alginolytic bacterium *Sphingomonas* sp. A1 [18]. A1-R was identified as an NADPH-dependent short-chain dehydrogenases/reductases (SDR)-superfamily enzyme [19]. Site-directed mutagenesis study indicated that the NADPH specificity of A1-R was attributable to the presence of a basic amino-acid residue Arg-39, which is responsible for the ionic binding to 2′-phosphate of NADPH [18]. On the other hand, an SDR-like enzyme gene *dehR* of an alginolytic bacterium *Vibrio splendidus* was suggested as preferring NADH to NADPH unlike A1-R [14]. However, detailed properties of this enzyme have not been reported yet. Although many SDR-superfamily enzymes are enrolled in databases [19], information about DEH reductases of alginolytic bacteria is still quite limited.

Previously, we reported on the isolation and characterization of the major alginate lyase FlAlyA from an alginate-assimilating bacterium *Flavobacterium* sp. strain UMI-01 [20]. FlAlyA degraded alginate in an endolytic manner to unsaturated di- and trisaccharide. We have recently found that crude extract from strain UMI-01 was capable of degrading alginate to unsaturated monosaccharide (see Experimental Section 4.7). This indicates that exolytic alginate lyase(s) is present in the crude extract along with FlAlyA. We will report on the enzymatic properties of this exolytic alginate lyase elsewhere. Furthermore, we performed genome analysis for strain UMI-01 and found an SDR-like enzyme gene located in the alginolytic gene cluster along with alginate lyase genes. This SDR-like enzyme gene, named *flred* in the present study, appeared to encode DEH reductase of strain UMI-01 because of its vicinal location to alginate-metabolic genes in the alginolytic operon.

In the present study, we report on the characteristics of the deduced amino-acid sequence of *flred* and basic properties of recombinant FlRed to enrich information about DEH reductases of alginolytic bacteria.

## 2. Results

### 2.1. Identification of FlRed Gene in Strain UMI-01 Genome

**Figure 1:**
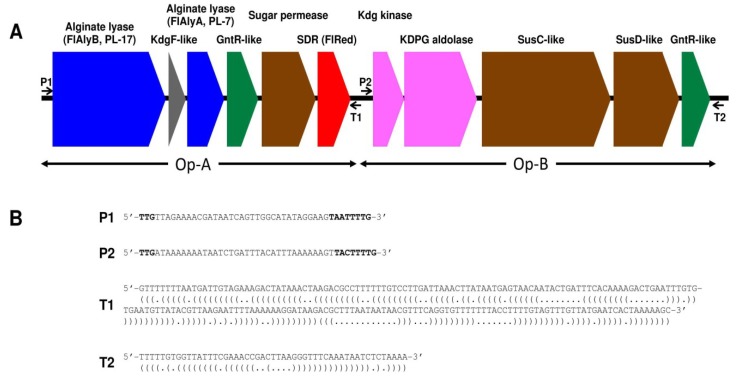
Schematic representation for an alginolytic gene cluster of *Flavobacterium* sp. UMI-01. (**A**) Organization of individual genes in the alginolytic gene cluster (15.6-kb cluster) of strain UMI-01. Genes are discriminated with different colors as follows. Blue, alginate lyase genes; gray, KdgF-like protein gene; green, transcription regulator genes; brown, membrane transportation genes; red, short-chain dehydrogenases/reductases (FlRed) gene *flred*; pink, KDG-metabolic enzyme genes. Locations for predicted promoters and terminators are indicated with arrows P1 and P2 and arrows T1 and T2, respectively; (**B**) Nucleotide sequences for predicted promoters P1 and P2 and terminators T1 and T2. The consensus promoter motif, -33 (TTG)/-7 (TAnnTTTG), in *Flavobacterium* sp. and *Bacteroides fragilis* proposed by Chen *et al.* [21,22] are shown with bold letters in P1 and P2. Parentheses under the T1 and T2 sequences indicate nucleotides predicted to form internal stem-loop structures.

**Figure 2:**
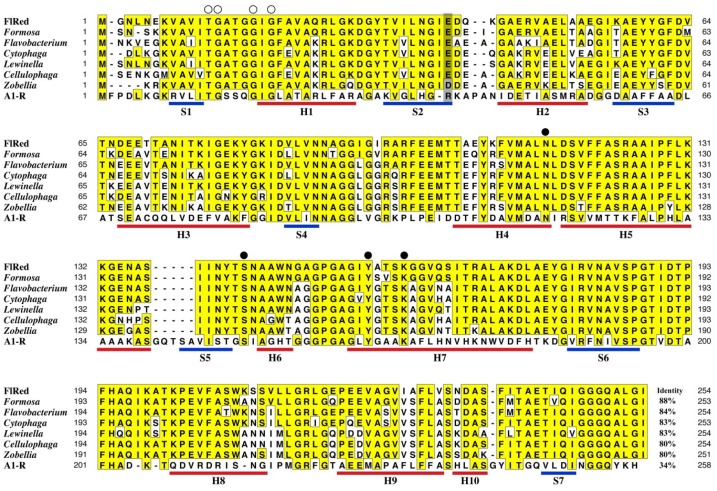
Comparison of amino-acid sequences between FlRed and other SDR-superfamily enzymes. Open circles show the residues conserved in the Rossmann fold cofactor-binding motif Thr-Gly-x-x-x-Gly-x-Gly. Closed circles represent the residues configuring catalytic tetrad. Gray box indicates the position corresponding to Arg39 of A1-R, which is responsible for NADPH specificity of A1-R. Blue and red lines represent the positions of β-sheets (S1–S7) and α-helices (H1–H10) in A1-R [18]. FlRed, DEH reductase from *Flavobacterium* sp. UMI-01 (present study); Formosa, acetoin (diacetyl) reductase from *Formosa agariphila* KMM 3901 (GenBank accession number, CDF80389); Flavobacterium, short-chain dehydrogenase from *Flavobacterium frigidarium* (GenBank accession number, WP_026707247); Cytophaga, short-chain dehydrogenase from *Cytophaga fermentans* (GenBank accession number, WP_027472031); Lewinella, short-chain dehydrogenase from *Lewinella cohaerens* (GenBank accession number, WP_020539555); Cellulophaga, short-chain dehydrogenase from *Cellulophaga algicola* (GenBank accession number WP_013552902); Zobellia, short-chain dehydrogenase from *Zobellia galactanivorans* (GenBank accession number, WP_013993961); A1-R, NADPH-dependent DEH reductase A1-R from *Sphingomonas* sp. A1 [18].

Figure 1 represents the schematic structure for an alginolytic gene cluster of 15.6 kbp found in strain UMI-01 genome (the nucleotide and deduced amino-acid sequences of individual genes are available from DDBJ, GenBank and EMBL with following accession numbers; FlAlyB, LC005508; KdgF-like protein, LC005509; FlAlyA [20], AB898059; GntR-like protein1, LC005510; sugar permiase, LC005511; SDR-like enzyme, LC005512; Kdg kinase, LC005513; KDPG aldolase, LC005514; SucC-like protein; LC005515; SusD-like protein, LC005516; GntR-like protein2, LC005517). The gene cluster comprised two putative operons, *i.e.*, Op-A and Op-B, which were defined by the occurrence of two sets of promoter and terminator sequences, *i.e.*, P1-T1 and P2-T2, which were predicted according to the consensus sequences proposed by Chen *et al.* [21,22]. Op-A comprised two alginate lyase genes (FlAlyA and FlAlyB genes), a KdgF-like protein gene, a GntR-like gene, a sugar permiase gene, and an SDR-like enzyme (FlRed) gene (*flred*), while Op-B comprised a Kdg-kinase gene, a KDPG-aldolase gene, a susC-like protein gene, a susD-like protein gene, and a GntR-like gene. Among these genes, the FlAlyA gene had been identified as the gene encoding the endolytic alginate lyase FlAlyA that belongs to polysaccharide-lyase family 7 (PL-7) [20]. KdgF-like protein gene was suggested to relate to pectin metabolism [23]; however, in this bacterium, it may participate in alginate metabolism. The SDR-like enzyme (FlRed) gene *flred* in Op-A was considered to be the DEH-reductase gene because of its involvement in the alginolytic operon. We then subjected the deduced amino-acid sequence of FlRed gene to BLAST search and retrieved some sequences of SDR-superfamily enzymes (Figure 2). The amino-acid identity between FlRed and the DEH reductase A1-R [18] was 34%, while the identities between FlRed and other SDR enzymes were 80%–88%. *Formosa* *agariphila* [24], *Zobellia galactanivorans* [25] and *Sphingomonas* sp. A1 [18] are known as alginate-assimilating bacteria, while *Cellulophaga algicola* [26], *Cytophaga fermentans* [27] *Flavobacterium frigidarium* (GenBank accession number, WP_026707247) and *Lewinella cohaerens* (GenBank accession number, WP_020539555) have not been identified as alginolytic bacteria. Specific sequence motifs of SDR-family enzymes, *i.e.*, the catalytic tetrad Asn-Tyr-Lys-Ser [28], the cofactor-binding sequence motif Thr-Gly-x-x-x-Gly-x-Gly in Rossmann fold were entirely conserved in FlRed as well as other SDR-superfamily enzymes, whereas, a basic amino-acid residue responsible for NADPH-specificity of *Sphingomonas* A1-R [18], *i.e.*, Arg39, was replaced by acidic residue Glu or Asp in FlRed and other SDR-superfamily enzymes. This suggested that FlRed was not NADPH-dependent but NADH-dependent unlike *Sphingomonas* A1-R.

### 2.2. Enzymatic Properties of Recombinant FlRed

**Figure 3:**
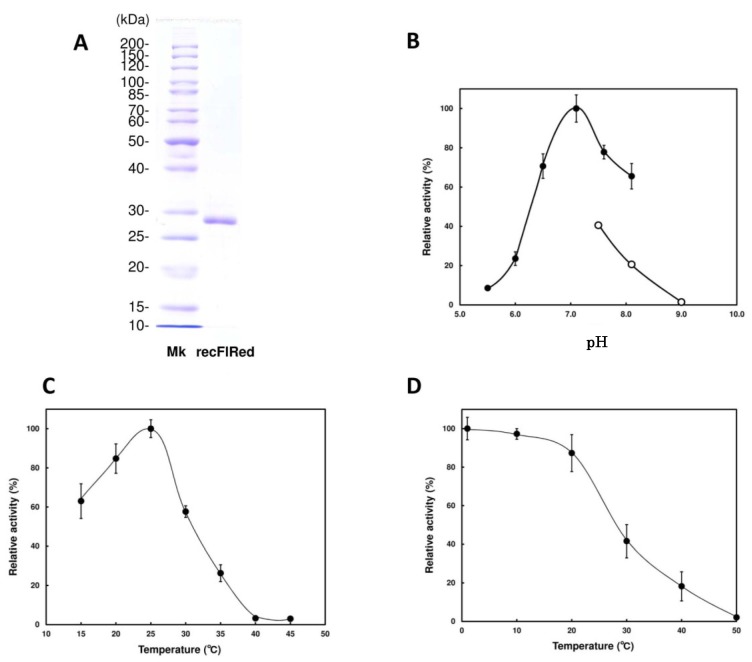
Effects of pH and temperature on the activity of recFlRed. (**A**) SDS-polyacrylamide gel electrophoresis for recFlRed. Mk, marker proteins; (**B**) pH dependence of recFlRed. Activity was measured in the reaction mixture adjusted to pH 5.5–8.5 as described under “Experimental Section.” (**C**,**D**) Temperature dependence and temperature stability of recFlRed. These measurements were also performed as described in the “Experimental Section.”

To examine if the translation product of *flred* exhibits the DEH-reductase activity, we produced recFlRed with an *Escherichia coli* expression system (see Experimental Section 4.3). As shown in Figure 3A, apparent molecular mass of recFlRed was estimated to be ~28 kDa by SDS-PAGE, which was consistent with the molecular mass calculated from the amino-acid sequence. The yield of recFlRed was considerably high, *i.e.*, ~40 mg of recFlRed could be produced in 1 L of *E. coli* culture. To determine co-factor specificity of recFlRed, the DEH-reducing activity was examined in the presence of either NADH or NADPH. As shown in Table 1, specific activities of recFlRed in the presence of NADH and NADPH were found to be 4.0 U/mg and 0.043 U/mg, respectively. This indicated that FlRed was a NADH-specific enzyme as predicted by the amino-acid sequence analysis. Optimal pH and temperature of recFlRed were observed at around 7.2 and 25 °C (Figure 3B,C), respectively. Thus, recFlRed was not so heat-stable, e.g., the activity decreased to half of the original level by the incubation at ~28 °C for 30 min (Figure 3D). Then, substrate specificity of recFlRed was examined with a variety of substrates such as aldehyde, ketone, keto ester, α-keto acid and aldose (Table 1). As a result, recFlRed was found to show appreciably no activity to such substrates. Accordingly, we concluded that recFlRed was the DEH reductase with high specificity to NADH.

**Table 1:** Substrate specificity of FlRed.

Substrates	Specific Activity (U/mg)	Relative Activity (%)
Aldehydes		
DEH		
(NADH)	4.0 ± 0.28	100
(NADPH)	0.043 ± 0.002	1.1
Benzaldehyde	N.D	N.D
Glutaraldehyde	N.D	N.D
*o*-Phthalaldehyde	N.D	N.D
Ketones		
4-Methyl-2-pentanone	N.D	N.D
2,5-Hexanedione	N.D	N.D
3-Chloropropiophenone	N.D	N.D
Ethylbenzoylacetate	N.D	N.D
Keto ester		
Methyl pyruvate	N.D	N.D
α-Keto acid		
α-Keto-glutaric acid	N.D	N.D
Aldose		
Glucose	N.D	N.D
Galactose	N.D	N.D

**Figure 4:**
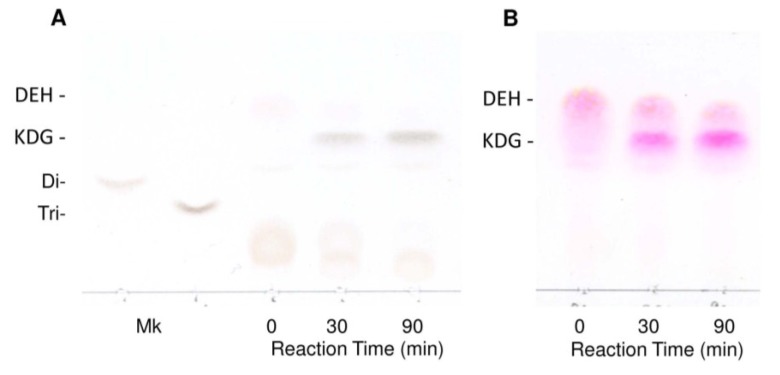

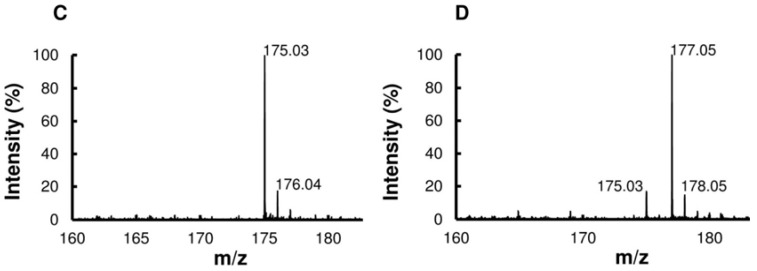
Analysis for the reaction products of DEH produced by recFlRed. The reaction was performed at 25 °C in a reaction mixture consisting of 10 mM DEH, 10 mM NADH, 10 mM potassium phosphate buffer (pH 7.0), 100 mM KCl, and 2.5 μg/mL recFlRed for 0–90 min. (**A**) Thin-layer chromatography for the products stained with 10% sulfuric acid in ethanol. Di- and Tri- indicate the standard disaccharide and trisaccharide produced by FlAlyA [20], respectively; (**B**) Thin-layer chromatography for the products stained with 4.5% thiobarbituric acid; (**C**) Mass spectrogram of DEH (*m*/*z* 175); (**D**) Mass spectrogram of the reaction product indicating the production of KDG (*m*/*z* 177).

The reaction products produced by recFlRed were then analyzed by thin-layer chromatography (TLC) and mass spectroscopy. As shown in Figure 4A, DEH was hardly detected on TLC by the sulfuric-acid detection; however, a clear band corresponding to KDG appeared in the reaction time at 30 and 90 min. Conversion of DEH to KDG was more definitely detected by thiobarbituric acid detection (Figure 4B). Namely, the original DEH band was gradually decreased with the extension of reaction time, while a KDG band with the mobility smaller than DEH concomitantly appeared. These results suggested that the DEH was converted to KDG by the action of recFlRed. The molecular masses of the DEH and the reaction product KDG were subsequently determined by MALDI-TOF-MS (Figure 4C,D). The peak with 175 *m*/*z* corresponding to DEH was detected before the reaction (Figure 4C), while this peak decreased by the reaction and instead a new peak with 177 *m*/*z* corresponding to KDG appeared (Figure 4D). These results supported the conversion of DEH to KDG by the action of recFlRed.

## 3. Discussion

Previously, we isolated and characterized the endolytic alginate lyase FlAlyA from an alginolytic *Flavobacterium* sp. strain UMI-01 [20]. Besides FlAlyA, exolytic alginate lyase(s) was also considered to be produced by this bacterium since the crude extract completely depolymerized alginate to an unsaturated monosaccharide, DEH (see Experimental Section 4.7). The DEH is generally considered to be metabolized via the specific pathway in some alginolytic bacteria [16,17,18]; however, the enzymes responsible for alginate metabolism had not been well characterized.

In the present study, we identified the DEH reductase gene *flred* in the *Flavobacterium* sp. strain UMI-01 genome. This gene was involved in the alginolytic operon along with other alginate-metabolic genes (Figure 1). Analysis for the deduced amino-acid sequence of *flred* indicated that the translation product FlRed comprised 254 residues that showed 34% and 80%–88% amino-acid identities to the DEH reductase A1-R from *Sphingomonas* sp. A1 and some bacterial SDR-like enzymes, respectively. In the early 1960s, the reduction of DEH to KDG in an alginolytic *Pseudomonas* sp. was reported to be catalyzed by a specific TPNH (NADPH)-linked dehydrogenase [16,17]. However, the properties of this enzyme had not been further investigated. Takase *et al.* recently succeeded in isolating the DEH reductase A1-R from *Sphingomonas* sp. A1 and identified it as a NADPH-dependent reductase belonging to SDR-superfamily [18]. Furthermore, they proposed that the basic residue Arg39 determined the NADPH specificity of A1-R and would bind 2′-phosphate moiety of NADPH. According to the multiple sequence alignment of A1-R and some SDR-superfamily enzymes, this residue was conserved among NADPH-dependent enzymes, while it was replaced by acidic residues in NADH-dependent enzymes [18]. In FlRed, the residue corresponding to Arg39 of A1-R was Glu (Glu39) (Figure 2). Similar replacement was also seen in other SDR-like enzymes. Residues neighboring Arg39 may also affect the electrostatic properties of this region. In this respect, Asp40 as well as Glu39 in FlRed was considered to be responsible for the co-factor specificity of this enzyme. By using recombinant FlRed, high specificity of this enzyme to NADH was confirmed (Table 1). Such NADH specificity was also reported in the SDR from an alginolytic bacterium *V. splendidus* [14]. However, detailed properties of *V. splendidus* DEH reductase have not been reported. Quite recently, Takase *et al.* [29] isolated another DEH reductase A1-R’ from *Sphingomonas* sp. A1. A1-R’ differed from A1-R in co-factor specificity, *i.e.*, it was specific to NADH. On the basis of X-ray analyses for A1-R and A1-R’, they concluded that the charged properties of two short and long loops, which surround co-factor binding region, structurally determine the co-factor specificity. Therefore, it should be considered that the co-factor specificity of DEH reductase is determined by the total properties of co-factor binding moiety including some specific charged residues. Although information about DEH reductases is still limited, we may conclude that at least two types of DEH reductases, *i.e.*, NADPH-dependent and NADH-dependent enzymes are present in alginolytic bacteria.

SDR-superfamily is known as the largest protein superfamily. The members are distantly related with 20%–30% amino-acid identity in pair-wise comparison [19]. Indeed, relatively low identity (34%) was found between FlRed and A1-R, although 80%–88% identity was found between FlRed and other SDR enzymes (Figure 2). The number of proteins classified under SDR superfamily went over 160,000 in 2012 and has been constantly increasing with the progress of large-scale genomic and metagenomic analyses [19]. Functions of SDR enzymes are known to be significantly deviated; however, reduction of DEH in alginate metabolic pathway had not been recognized as an SDR function. Thus, DEH-reductases of alginolytic bacteria will be attractive materials in the studies on substrate specificity and molecular evolution of SDR-superfamily enzymes.

Finally, we should mention the high reaction efficiency of recFlRed. Namely, ~90% of DEH was found to be converted to KDG by recFlRed in the presence of stoichiometric amount of NADH (data not shown but see Figure 4). Since the yield of DEH from alginate was ~70% (see “Experimental Section”) and the yield of recFlRed was ~40 mg from 1 L culture, KDG will be easily produced in high yield by this system. KDG is the essential substrate for the investigation of KDG metabolic enzymes such as KDG kinase and KDGP aldolase. The KDG-producing system established in the present study will provide sufficient amount of KDG from alginate and expand the studies on alginate metabolism.

## 4. Experimental Section

### 4.1. Materials

*Flavobacterium* sp. strain UMI-01 was aerobically cultivated at 30 °C for 24 h in a 1% (w/v) alginate medium as described previously [20]. Sodium alginate (*Macrocystis pyrifera* origin) was purchased from Sigma-Aldrich (Louis, MO, USA), Bacto Trypton and Yeast Extract were from Becton and Dickinson (Sparks, MD, USA). pTac-1 cloning vector and pCold I expression vector along with respective host strains *E. coli* DH5 α and BL21(DE3) were from BioDynamics (Tokyo, Japan) and TaKaRa (Shiga, Japan), respectively. Ni-NTA resin was purchased from Qiagen (Hilden, Germany), and TOYOPEARL SuperQ-650S was from Toyo soda Mfg, Co. (Tokyo, Japan). TLC Silica gel 60 plates were purchased from Merck KGaA (Darmstadt, Germany). NADH, NADPH, benzaldehyde, glutalaldehyde, glyceraldehyde, o-phthalaldehyde, 4-methyl-2-pentanone, 2,5-hexanedione, 3-chloropropopiophenone, ethylbenzoylacetate, methyl pyruvate, α-ketoglutaric acid, glucose, galactose and other chemicals were from Wako Pure Chemical Industries, Ltd. (Tokyo, Japan).

### 4.2. Genome Analysis for Flavobacterium sp. Strain UMI-01

Total DNA from strain UMI-01 was prepared with an ISOHAIR DNA extraction kit (Nippon Gene, Tokyo, Japan) from 1.0 g (wet weight) of bacterial pellet. Approximately 15 μg of the total DNA was subjected to genomic analysis with an Illumina Hiseq X sequencer (Illumina, Inc., San Diego, CA, USA) in Hokkaido System Science (Sapporo, Japan). Draft genome sequence was annotated by MiGAP (http://www.migap.org) and a total of 31 contigs comprising 1274~716,448 bp were assigned. An SDR-like enzyme gene, *flred*, was found in an alginolytic operon along with the endolytic alginate lyase FlAlyA gene (see Figure 1). This operon located in the contig 7 comprising 205,520 bp that includes 207 coding regions. Total genome structure for strain UMI-01 will be published elsewhere.

### 4.3. Production of Recombinant FlRed

**Figure 5:**
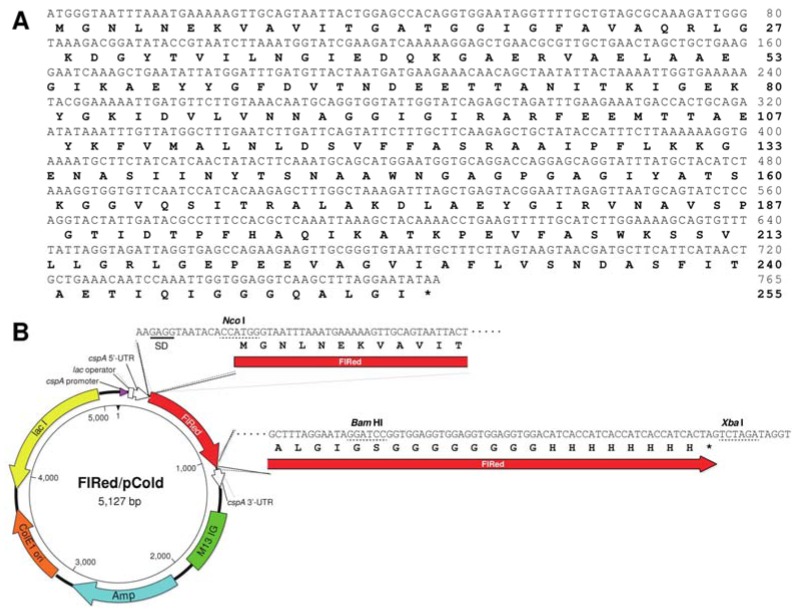
Nucleotide and deduced amino-acid sequences of *flred* and the structure of pCold I vector. (**A**) Nucleotide and deduced amino-acid sequences of *flred*; (**B**) Structure of pCold I vector for the expression of recFlRed.

Coding region of *flred* gene was amplified by genomic PCR using specific forward and reverse primers, 5′-GTAGTAAATAATTAATTTAGAATAAAG-3′ and 5′-AGTTTAATCAAGGACAAAAAAGGCGTC-3′, respectively, which were synthesized on the basis of 5′- and 3′-flanking sequences of *flred* (see Figure 1). PCR was performed in 50 μL of reaction mixture containing 10 ng of total DNA and 1 µM each primer using Phusion^®^ Hot Start Flex 2× Master Mix (New England Biolabs, Ipswich, MA, USA). After preheating at 98 °C for 2 min, the reaction of 98 °C for 10 s, 50 °C for 15 s, and 72 °C for 30 s was repeated 30 cycles. The PCR product was treated with A-attachment mix (Toyobo, Osaka, Japan) and ligated to pTac-1. The nucleotide sequence of the cloned DNA in pTac-1 was analyzed with a BigDye-Terminator Cycle Sequence kit (Applied Biosystems, Foster City, CA, USA) and a DNA sequencer 3130xl (Applied Biosystems, Foster, CA, USA). Restriction sites were introduced to the 5′- and 3′-termini of the cloned *flred* using the primers, 5′-AGGTAATACACCATGGGTAATTTAAATGAAAAAGTTG-3′ (*Nco* I site was underlined) and 5′-CACCTCCACCGGATCCTATTCCTAAAGCTTGACCTCC-3′ (*Bam*H I site was underlined). The amplified DNA was ligated to pCold vector digested by *Nco* I and *Bam*H I using In-Fusion system (Clontech Laboratories, Mountain View, CA, USA). The pCold I vector had been modified to add 8×Gly+8×His-tag to the C-terminus of recombinant protein and to remove original *N*-terminal translation enhancing element, His-tag and Factor Xa recognition site as reported previously [20]. The nucleotide and deduced amino-acid sequences of *flred* and the circular map for the expression pCold I vector are shown in Figure 5A,B. The resultant plasmid was introduced to *E. coli* BL21 (DE3), and it was cultured at 37 °C overnight in 2xYT medium. To express recombinant FlRed (recFlRed), 0.1 mM IPTG was added to the culture and incubated at 15 °C for 12 h. Bacterial cells were harvested by centrifugation at 5000× *g* for 15 min and homogenized by sonication in a buffer containing 10 mM imidazole-HCl (pH 8.0), 0.5 M NaCl, 1% Triton X-100, and 0.01 mg/mL lysozyme. The homogenate was centrifuged at 10,000× *g* for 15 min, and recFlRed in the supernatant was adsorbed to Ni-NTA resin in a conical tube with gentle mixing. After the incubation for 30 min on ice, resin was set in a disposable plastic column and washed with a buffer containing 30 mM imidazole-HCl (pH 8.0)–0.5 M NaCl. recFlRed was eluted with 150 mM imidazole-HCl (pH 8.0)–0.5 M NaCl from the column and dialyzed against 0.1 M NaCl–10 mM sodium phosphate buffer (pH 7.5) before use.

### 4.4. Sodium Dodecyl Sulfate-Polyacrylamide Gel Electrophoresis

Sodium dodecyl sulfate (SDS) polyacrylamide-gel electrophoresis was performed according to the method of Porzio and Pearson [30] using 10% polyacrylamide containing 0.1% SDS. After the electrophoresis, the gel was stained with 0.1% Coomassie Brilliant Blue R-250 in 50% methanol–10% acetic acid and the background of the gel was destained with 5% methanol–7% acetic acid. Protein Ladder (10–250 kDa, New England Biolabs, Ipswich, MA, USA) was used as a molecular mass marker.

### 4.5. Determination of Protein Concentration

Protein concentration was determined by the biuret method [31] or the method of Lowry *et al*. [32] using bovine serum albumin fraction V as a standard protein.

### 4.6. Assay for Alginate Lyase Activity

Alginate lyase activity was assayed in a reaction mixture containing 0.15% sodium alginate and 10 mM sodium phosphate (pH 7.0) at 30 °C. The activity was determined by measuring the increase in Abs_235nm_ due to the formation of unsaturated sugar in the non-reducing terminus of split site. One unit of alginate lyase activity was defined as the amount of enzyme that increases Abs_235nm_ to 0.01 for 1 min reaction.

### 4.7. Preparation of Crude Extract from Strain UMI-01

**Figure 6:**
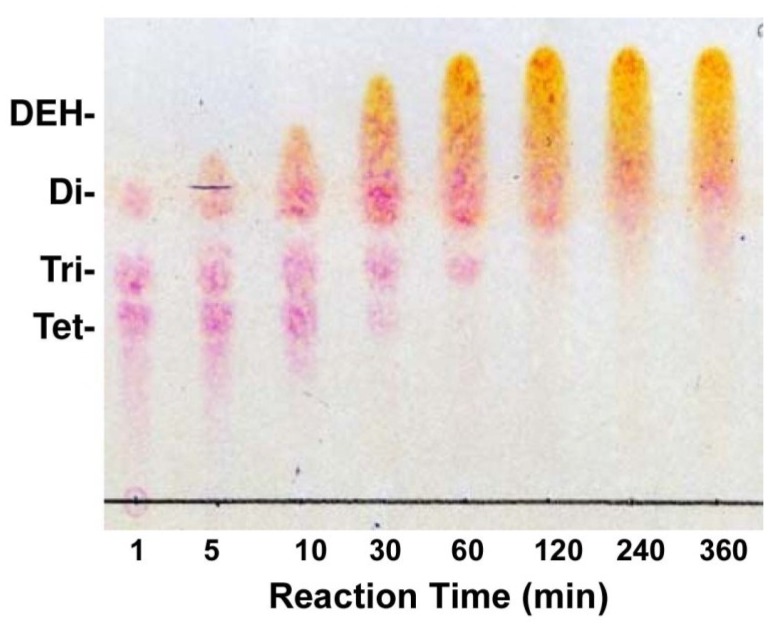
Production of DEH by crude enzyme from strain UMI-01. An aliquot (0.1 mL) was withdrawn from the reaction mixture containing 1.5% (w/v) alginate and 100 U/mL of alginate lyases from strain UMI-01 at appropriate time and 2 μL of each sample was subjected to TLC plate. The degradation products were detected by 4.5% thiobarbituric acid staining after periodic acid treatment.

*Flavobacterium* sp. strain UMI-01 cultured in 1 L of a minimum salt medium containing 1% sodium alginate (OD_600nm_ ~1.5) was harvested by centrifugation at 4600 × *g* for 30 min and suspended with 20 mL of 10 mM sodium phosphate (pH 7.0). The suspension was centrifuged again and the precipitates were suspended with 10 mL of the same buffer. The suspension was frozen at −20 °C overnight and thawed at 15 °C. The suspension was then sonicated with an ULTRASONIC homogenizer VP-050 (TAITEC, Saitama, Japan) at 20 kHz-25 W and 15 s × 10 times on ice. The homogenate was centrifuged at 10,000× *g* for 15 min. The supernatant was dialyzed against 2 mM sodium phosphate buffer (pH 7.0) for 24 h with the dialysis tube (12,000-Da cut off, Sigma-Aldrich, St. Louis, MO, USA) to remove low molecular size components. By this procedure, the crude extract containing ~20,000 U of alginate lyases was obtained. The crude extract could degrade alginate to unsaturated monosaccharide (Figure 6) indicating the occurrence of both endolytic and exolytic alginate lyase(s) in the extract.

### 4.8. Preparation of DEH

DEH was prepared by the digestion of alginate with the crude extract from strain UMI-01 as follows. A total of 15 g of sodium alginate was dissolved in 100 mL of 2 mM sodium phosphate (pH 7.0) and the crude extract was added to the mixture to make 100 U/mL of alginate lyases. The enzyme reaction was carried out at 30 °C for 12 h and terminated by the addition of 1 M acetic acid to make final concentration 20 mM. The precipitates formed were spun down at 10,000× *g* for 15 min and the supernatant containing DEH and small amount of disaccharide was subjected to a column of TOYOPEARL SuperQ (2.5 × 30 cm) pre-equilibrated with 10 mM acetic acid. The column was washed with 300 mL of distilled water and the materials adsorbed to the column were eluted by the linear gradient of 0–0.2 M NaCl in water (total 1 L). The eluent was collected as 15 mL fractions and elution of DEH and disaccharide was monitored by the phenol–sulfuric acid method [33] and TLC analysis. In this chromatography, DEH and disaccharide were eluted at around 0.1 M NaCl and 0.17 M NaCl, respectively. A fraction containing ~20 mg/mL of DEH was used as the substrate solution for DEH-reductase assay. Contamination of proteins and nucleic acids in the DEH preparation was negligible.

### 4.9. Assay for DEH-Reducing Activity

DEH-reducing activity of recFlRed was assayed at 25 °C with a standard reaction mixture containing 2.5 mM DEH, 100 mM KCl, 10 mM potassium phosphate (pH 7.0), 0.2 mM NADH or NADPH, and an appropriate amount of enzyme. The progress of reaction was monitored by measuring the decrease in the absorbance at 340 nm due to the oxidation of NADH or NADPH. One unit (U) of reductase activity was defined as the amount of enzyme that produces 1 μmol of reduced product per min. pH dependence of recFlRed was determined in reaction mixtures adjusted to pH 5.5–8.5 with 20 mM potassium phosphate buffer, and pH 7.5–9.0 with 20 mM glycine-NaOH buffer. Temperature dependence was determined at 15–45 °C in the standard reaction mixture. Thermal stability of recFlRed was assessed by measuring the activity remained after the heat treatment at 2–50 °C for 30 min in the standard reaction mixture. The mean activity values for three different assays were shown with standard deviations.

### 4.10. Assessment of Substrate Specificity of RecFlred

Substrate specificity of recFlRed was assessed by using following substrates: DEH, benzaldehyde, glutalaldehyde, glyceraldehyde, *O*-phthalaldehyde, 4-methyl-2-pentanone, 2,5-hexanedione, 3-chloropropopiophenone, ethylbenzoylacetate, methyl pyruvate, α-keto-glutaric acid, glucose, galactose. These substrates were added to the reaction mixture to make final concentration 2.5 mM.

### 4.11. Thin-Layer Chromatography of Reaction Products

Reaction products of FlRed were analyzed by thin-layer chromatography (TLC) using TLC Silica gel 60 plates (Merck KGaA, Darmstadt, Germany). Approximately 5 μL of reaction products were taken out from the reaction mixture at various times and applied to the TLC plate. Development of TLC was performed with 1-butanol:acetic acid:water = 2:1:1 (v:v:v) and the sugars on the plate were detected with either 10% sulfuric acid staining or 4.5% thiobarbituric acid staining after periodic acid treatment.

### 4.12. Mass Spectrometry for DEH and KDG

Molecular masses of DEH and KDG were analyzed with a matrix-assisted laser desorption ionization-time of flight mass spectrometry (MALDI-TOF MS) (Proteomics Analyzer 4700, Applied Biosystems, Foster City, CA, USA). 1 μL of reaction mixture containing DEH and/or KDG was mixed with 1 μL of 10 mg/mL 2,5-dihydroxybenzoic acid (Sigma-Aldrich Japan, Tokyo, Japan) in acetonitrile and applied to a sample plate. After drying *in vacuo*, molecular masses of samples were determined in a negative ion mode.

## 5. Conclusions

In the present study, the DEH reductase gene *flred* was successfully identified in the genome of an alginolytic bacterium *Flavobacterium* sp. strain UMI-01. According to the analysis for the deduced amino-acid sequence of *flred*, the translational product FlRed was regarded as an SDR-superfamily enzyme. FlRed was considered to prefer NADH to NADPH by the comparison of the amino-acid sequences of SDR enzymes. Enzymatic properties of FlRed were investigated with recombinant enzyme produced by an *E. coli* expression system. RecFlRed could be produced in a yield of 40 mg/L culture and showed high specificities to DEH and NADH. This enzyme seems to be useful for the production of KDG that is essential for the studies of alginate metabolic pathway in alginolytic bacteria.
